# Synthesis of Fibrin-Type I Collagen Biomaterials via an Acidic Gel

**DOI:** 10.3390/molecules27072099

**Published:** 2022-03-24

**Authors:** Kun Wang, Marie Camman, Gervaise Mosser, Bernard Haye, Léa Trichet, Thibaud Coradin

**Affiliations:** Sorbonne Université, CNRS, Laboratoire de Chimie de la Matière Condensée de Paris, 75005 Paris, France; kunwangfighting@gmail.com (K.W.); marie.camman@sorbonne-universite.fr (M.C.); gervaise.mosser@sorbonne-universite.fr (G.M.); bernard.haye@sorbonne-universite.fr (B.H.); lea.trichet@sorbonne-universite.fr (L.T.)

**Keywords:** hydrogels, fibrin, collagen

## Abstract

Fibrin-Type I collagen composite gels have been widely studied as biomaterials, in which both networks are usually formed simultaneously at a neutral pH. Here, we describe a new protocol in which mixed concentrated solutions of collagen and fibrinogen were first incubated at acidic pH to induce fibrinogen gel formation, followed by a pH change to neutral inducing collagen fiber formation. Thrombin was then added to form fibrin-collagen networks. Using this protocol, mixed gels containing 20 mg.mL^−1^ fibrin and up to 10 mg.mL^−1^ collagen could be prepared. Macroscopic observations evidenced that increasing the content of collagen increases the turbidity of the gels and decreases their shrinkage during the fibrinogen-to-fibrin conversion. The presence of collagen had a minor influence on the rheological properties of the gels. Electron microscopy allowed for observation of collagen fibers within the fibrin network. 2D cultures of C2C12 myoblasts on mixed gels revealed that the presence of collagen favors proliferation and local alignment of the cells. However, it interferes with cell differentiation and myotube formation, suggesting that further control of in-gel collagen self-assembly is required to elaborate fully functional biomaterials.

## 1. Introduction

After injury, the vascular wall is ruptured, and fibrinogen reacts with thrombin to form a fibrin clot that entraps red blood cells and activated platelets [1]. There are many factors influencing the formation of fibrin clots, such as protein and enzyme concentration, pH, temperature, ionic strength and calcium amount [2,3]. Factor XIII provides covalent bonds between chains of adjacent fibrin molecules, which increase the fiber thickness, promote red blood cell retention and slows down clot lysis [4]. In vitro, in the absence of factor XIII, fibrin hydrogels usually show fast biodegradability and low storage modulus values [5,6]. There are many approaches to solve these problems, such as increasing fibrinogen and thrombin concentration [7,8], adding chemical cross-linkers [9,10] or mixing fibrinogen with particles or other polymers to form composite hydrogels [11,12,13,14,15,16].

Type I collagen is the major component of most extracellular matrices and plays an important role in the design of biomaterials [17,18,19,20]. Its mechanical properties and rate of biodegradation can be tuned to optimize its application in tissue engineering [21,22,23,24]. Fibrin-type I collagen hydrogels have already been well studied and have shown promising applications in tissue engineering, especially in the cardiovascular area [25,26,27,28,29]. Several methods have been developed to prepare fibrin-collagen hydrogels starting from protein mixtures [30]. In most cases, collagen, fibrinogen and thrombin are mixed at low temperature in the presence of a neutralizing medium (a base or a buffer), and gel formation is achieved after incubation at 37 °C. However, the two protein networks grow almost independently and establish only weak interactions within the mixed structure [31]. Moreover, such a protocol is not compatible with the use of solutions of high concentrations of collagen that are very viscous and, therefore, difficult to neutralize homogeneously by solution routes [32]. 

Here, looking for an alternative synthetic route to such mixed fibrin-collagen hydrogels, we turned our attention to the reported ability of fibrinogen alone (i.e., without thrombin addition) to form transparent gels at acidic pH and 37 °C [33]. It was shown that these gels could be further converted into fibrin gels after neutralization and the addition of thrombin [33]. On this basis, we decided to take advantage of this process to design fibrin-collagen hydrogels through a new protocol in which collagen and fibrinogen are first mixed and incubated in acidic pH to allow fibrinogen gel formation, and then a change of pH to neutral is used to induce collagen gel formation. Finally, fibrinogen to fibrin conversion is achieved upon thrombin addition. We show that the resulting composite hydrogels consist of a porous fibrin network incorporating collagen fibers, which have a minor influence on the rheological properties of the gels. 2D cultures of C2C12 myoblasts suggest that these cells are able to respond to the presence of collagen domains, favoring their local alignment and overall proliferation. However, the mixed gels show poor ability to promote myotube formation, calling for better control of collagen self-assembly within the fibrinogen matrix.

## 2. Results

**Figure 1:**
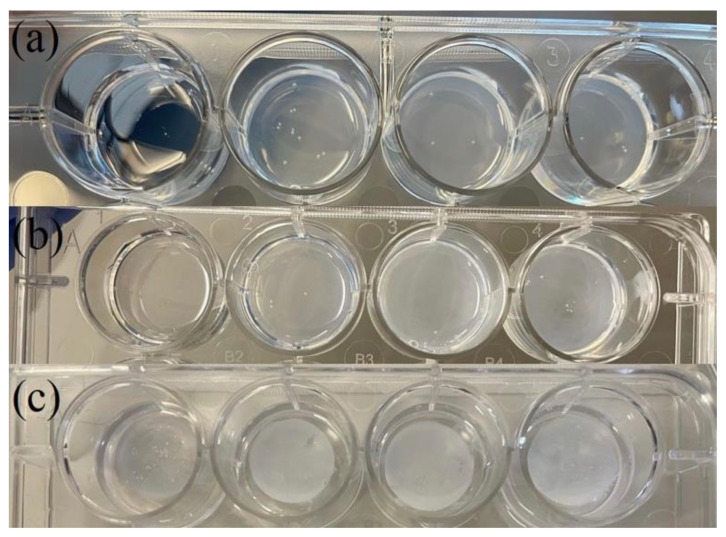
Macroscopic images of at the different stages of the synthetic process: (**a**) acidic mixture (pH 3.6) left at 37 °C overnight, (**b**) after washing with citrate solution (pH 7.2) to neutral pH and (**c**) after incubation with thrombin in citrate at 37 °C overnight. Final concentrations: fibrinogen = 20 mg.mL^−1^, collagen (from left to right) = 0, 2.5, 5, 10 mg.mL^−1^.

Macroscopic images of gel morphology at the different stages of the process are shown in Figure 1. In the first step, fibrinogen solutions (final concentration = 20 mg.mL^−1^) mixed with collagen solutions (final concentration 2.5, 5, or 10 mg.mL^−1^) or acetic acid buffer at pH 3.6 were prepared and left at 37 °C overnight. All samples formed gels whose turbidity increased with collagen content (Figure 1a). After washing with citrate buffer (pH 7.2), fibrin-collagen gels became even whiter (Figure 1b). After incubation in the presence of thrombin at 37 °C overnight, all gels underwent a significant shrinkage, as shown in Figure 1c. Noticeably, such a shrinkage decreased as collagen concentration increased. 

**Figure 2:**
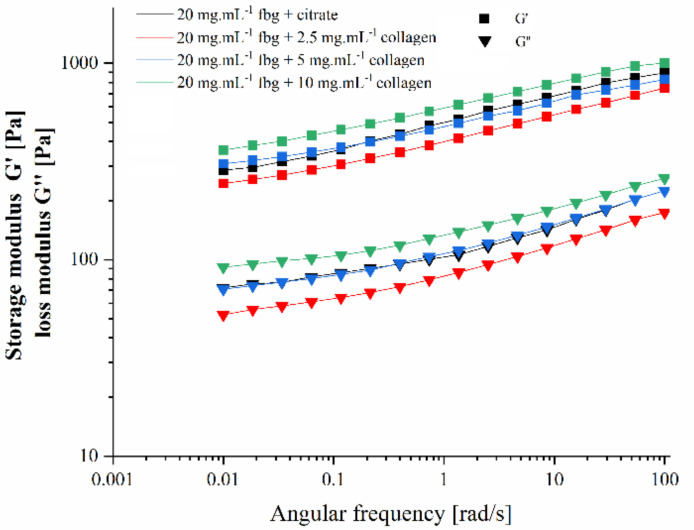
Variation of storage modulus G′ and loss modulus G″ with angular frequency of the final fibrin and fibrin-collagen hydrogels.

Rheological properties of hydrogels were measured using frequency sweep from 0.01 to 100 rad.s^−1^ with 1% amplitude. All gels exhibited an increase in storage modulus with increasing frequency (Figure 2). In the presence of thrombin, the addition of 2.5 mg.mL^−1^ collagen decreased the storage modulus G′ compared to pure fibrin. There was no obvious difference between pure fibrin and after 5 mg.mL^−1^ collagen addition, while 10 mg.mL^−1^ collagen slightly increased the G′ value compared to pure fibrin. The loss modulus G″ showed similar evolution as G′.

**Figure 3:**
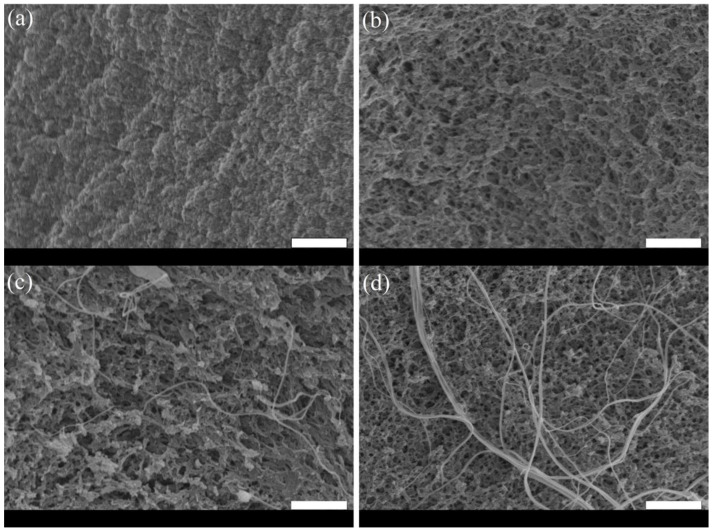
SEM images of the fibrin and fibrin-collagen hydrogels: (**a**) pure fibrin at 20 mg.mL^−1^; (**b**) fibrin 20 mg.mL^−1^:collagen 2.5 mg.mL^−1^; (**c**) fibrin 20 mg.mL^−1^:collagen 5 mg.mL^−1^; (**d**) fibrinogen 20 mg.mL^−1^:collagen 10 mg.mL^−1^. (scale bar = 2 μm).

**Figure 4:**
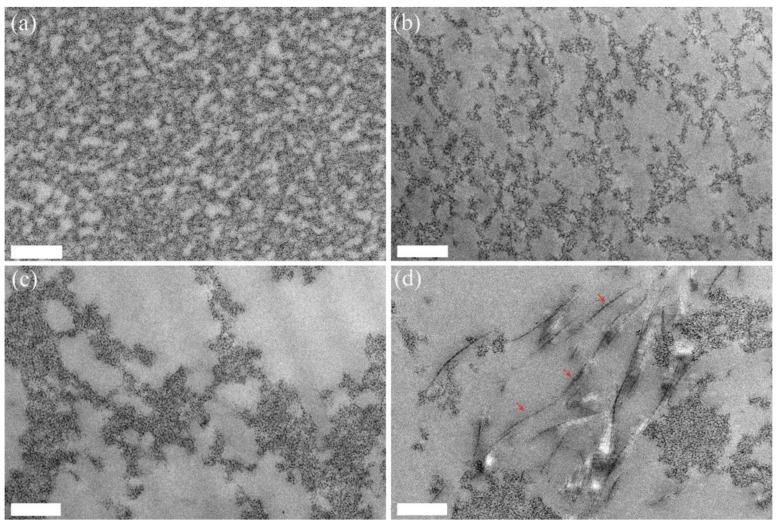
TEM images of the fibrin and fibrin-collagen hydrogels: (**a**) pure fibrin at 20 mg.mL^−1^; (**b**) fibrin 20 mg.mL^−1^:collagen 2.5 mg.mL^−1^; (**c**) fibrin 20 mg.mL^−1^:collagen 5 mg.mL^−1^; (**d**) fibrinogen 20 mg.mL^−1^:collagen 10 mg.mL^−1^. (Scale bar = 500 nm). Red arrows indicate collagen fibrils.

The structure of hydrogels was studied by scanning electron microscopy (SEM) (Figure 3) and transmission electron microscopy (Figure 4). From SEM images, the network obtained for pure fibrin solution looked compact (Figure 3a). Upon collagen addition, the network became porous, and collagen fibers were easily evidenced at 5 mg.mL^−1^ and 10 mg.mL^−1^ collagen concentrations (Figure 3b–d). 

TEM images showed that, in the absence of collagen, the gel obtained for pure fibrin consisted of well-distributed, closely packed small fibers (Figure 4a). With an increasing amount of collagen, the background network became more and more porous, and the observation of large fibers exhibiting the characteristic D-banding patterns of collagen were clearly observed for the highest collagen content (Figure 4b–d).

**Figure 5:**
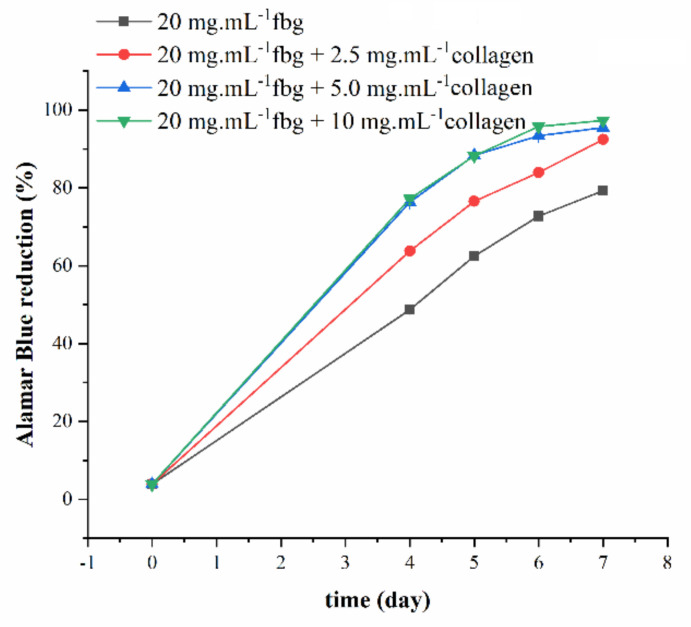
Proliferation of C2C12 cells on fibrin and fibrin-collagen composite gels as monitored by Alamar Blue test.

The first evaluation of the impact of collagen on the ability of fibrin gels to act as a 2D substrate for muscular cell development was performed. C2C12 myoblast cells were seeded on fibrin and fibrin-collagen gels, and their proliferation was followed by an Alamar Blue test. Cells grew rapidly in the first four days on all hydrogels, but proliferation was the highest for fibrin-collagen gels containing 5 and 10 mg.mL^−1^ collagen and the lowest in the absence of collagen, with an intermediate value for 2.5 mg.mL^−1^ collagen (Figure 5). For the two first samples, a plateau was reached on the sixth day as all the Alamar Blue reagent had been reduced. For the two other samples, growth continued over 1 week but was always greater on gels containing 2.5 mg.mL^−1^ collagen than on pure fibrin gels.

**Figure 6:**
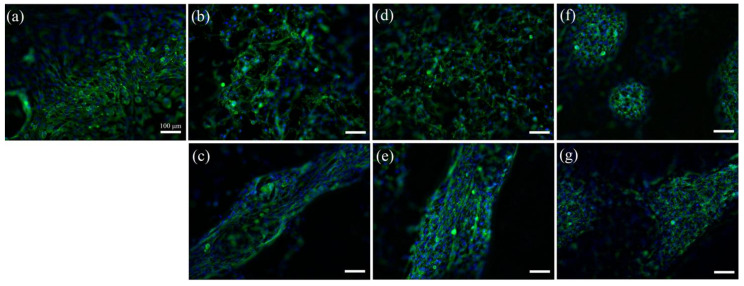
Fluorescence images of C2C12 cells cultured for 6 days in serum-rich medium on (**a**) 20 mg.mL^−1^ fibrin; (**b**,**c**) 20 mg.mL^−1^ fibrin with 2.5 mg.mL^−1^ collagen (**d**,**e**) 20 mg.mL^−1^ fibrin and 5 mg.mL^−1^ collagen; (**f**,**g**) 20 mg.mL^−1^ fibrin with 10 mg.mL^−1^ collagen. (Blue: DAPI, green: Alexa Fluor 488 Phalloidin). (Scale bar = 100 μm).

In order to study cell morphology and distribution on the gel surface, cells were stained with 4′,6-diamidino-2-phenylindole (DAPI) and Alexa Fluor 488 Phalloidin for visualization of nuclei and actin, respectively, on day 6 (Figure 6). Cells were evenly distributed on the pure fibrin gel surface (Figure 6a). In the presence of collagen, adhering cells were distributed into two distinct populations, one reminiscent of the pure fibrin substrate (Figure 6b,d,f) while the other adopted a fiber-like organization (Figure 6c,e,g). With increasing collagen content, these fibers grew in diameter and then exhibited a branched organization.

**Figure 7:**
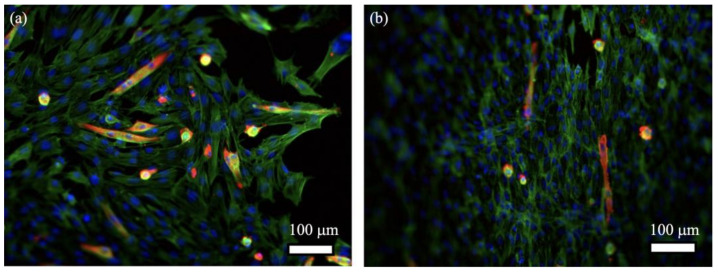
Fluorescence images of C2C12 cells cultured for 4 days in serum-rich and 7 days in low-serum DMEM on (**a**) 20 mg.mL^−1^ fibrin; (**b**) 20 mg.mL^−1^ fibrin with 10 mg.mL^−1^ collagen (Blue: DAPI, green: Alexa Fluor 488 Phalloidin, red: MF20- Alexa Fluor 546).

Finally, the differentiation of C2C12 cells growing on the hydrogels was evaluated. Myotubes were evidenced by MF20 staining. For pure fibrin gels, myotubes could be observed, but they were rather few and short. In the presence of 10 mg.mL^−1^ collagen, myotubes were only sparingly identified (Figure 7).

## 3. Discussion

Biopolymer-based hydrogels have found a wide range of applications, especially in the medical, food and analytical fields [34,35,36]. In particular, fibrin and type I collagen are two key proteins in the human body and have therefore been widely used for the design of biomaterials [6,20]. Their combination within mixed hydrogels has also been well-described as these two proteins exhibit some complimentary properties [30]. Among the described protocols, the most common is based on the mixing of collagen, thrombin and fibrinogen in acidic conditions at 4 °C, followed by neutralization and incubation at 37 °C [25,26,27]. However, such protocols were so far used for relatively low concentrated protein solutions (i.e., <10 mg.mL^−1^) [30], most likely because of the high viscosities of concentrated collagen solutions at low temperatures [32]. The recently-reported protocol that forms fibrinogen gels in acidic conditions and without thrombin addition seemed more amenable to the prepared mixed gels with high contents of both proteins [33]. 

**Scheme 1:**
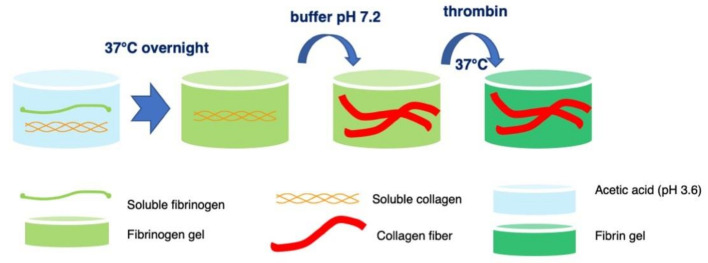
Overview of the synthetic pathway to fibrin-collagen hydrogels.

The different steps of our new synthetic pathway to elaborate fibrin-collagen mixed hydrogels are shown in Scheme 1. Fibrinogen and collagen acidic solutions were mixed first. Upon incubation at 37 °C, a fibrinogen gel was formed, whereas collagen should remain in a triple helix form. Rinsing with a neutral buffer should further allow for collagen self-assembly. Finally, the addition of thrombin would lead to fibrinogen to fibrin conversion without impacting the collagen network. Macroscopic images are in good agreement with this process. In particular, the transition from transparent to turbid gels upon neutralization, which is all the more marked as collagen concentration is high, supports the hypothesis that collagen self-assembly occurs within the fibrinogen network. Moreover, the observed shrinkage upon thrombin addition confirms that fibrinogen is still able to react with this enzyme. 

SEM and TEM images both confirm that collagen fibers were formed within the fibrin gels. However, they also demonstrate that the presence of collagen impacts the fibrin network, increasing the average pore size from ca. 100 nm in the absence of collagen to more than 500 nm in the presence of 5 mg.mL^−1^ collagen. This agrees with the observation that gel shrinkage is the highest in the absence of collagen and decreases as collagen content increases. Whereas a decrease in rheological properties would have been expected with increasing porosity (i.e., decreasing density), only the smallest collagen concentration (2.5 mg.mL^−1^) led to a decrease in the G′ and G″ values compared to pure fibrin gels, whereas a higher content (5 and 10 mg.mL^−1^) led to a small but quantifiable increase of these properties. The most plausible explanation is that collagen fibers may act as reinforcing elements for the fibrin network and can compensate for increasing porosity when present in sufficient amounts. The collagen-induced increase in the fibrin network porosity is difficult to explain at this time. However, it must be noticed that the initial fibrinogen gel structure is formed in the presence of collagen triple helices. Since both proteins are positively charged in acidic conditions, it is very likely that collagen molecules are located within the porosity of the gel, following a phase separation model. Upon neutralization, collagen molecules must diffuse through the porous network and assemble collectively to form fibrils, leaving empty (i.e., collagen-free) pores. This hypothesis is strengthened by the TEM image of the gels containing 10 mg.mL^−1^ collagen (Figure 4d), showing that collagen fibers tend to be clustered together rather individually dispersed in the fibrin network porosity. Noticeably, although the process by which such a clustering occurs is different, this result is reminiscent of the formation of collagen domains dispersed in fibrin matrices observed upon neutralization of collagen-fibrinogen-thrombin mixtures [27,31]. 

To obtain a first evaluation of the potentialities of these mixed hydrogels as biomaterials, they were used as substrates for the 2D culture of C2C12 myoblasts. These cells were selected because type I collagen, as the major component of the extracellular matrix in the perimysium and epimysium of muscle tissue, plays a significant role in the regulation of myoblast behaviors [37]. In particular, hydroxyprolyl-glycine, a collagen-derived dipeptide, promotes C2C12 myoblast fusion and myotube hypertrophy [38]. In line with our expectations, the growth rate of the seeded C2C12 increased with collagen content in the hydrogels. In addition, fluorescence imaging revealed that these cells could locally grow following a fiber-like organization. Indeed, it is not possible to make a direct correlation between the collagen fibers observed by SEM, that are below 1 μm in diameter, and the extent of these cell structures over several microns. However, it is possible to assume that the alignment of the C2C12 reflects the presence of collagen-rich domains on the gel surface, as already observed on collagen threads [39]. It must also be pointed out that variations in pore size should also impact cell adhesion and proliferation [40].

In a further step, the ability of seeded cells to differentiate and form myotubes was studied. It was noticed that pure fibrin gel obtained by the acidic route yields to fewer and shorter myotubes compared to fibrin gels obtained using the common neutral pH route, although C2C12 proliferation rates were similar for both types of gels [13]. This points out that, although the acidic pathway does allow obtaining fibrin networks, they have distinct structures and properties from common ones [33]. These differences may impact the ability of myoblasts to contract and remodel the fibrin network, a key requirement for myotube formation [41]. Furthermore, the presence of collagen seems not to be beneficial to myotube formation. Attempts to evaluate the myotube fusion index, i.e., the fraction of nuclei incorporated in myotubes [42], for mixed gels were unsuccessful due to the low occurrence of MF20-stained cells. The influence of type I collagen on myoblasts differentiation has been reported on several occasions, pointing out that it can have either an inhibitory or a promoting effect, depending strongly on collagen organization and fiber size [43,44,45]. In particular, it was reported that myoblasts grown on fibrin-collagen hydrogels could form myotubes on flat substrates but not on threads, evidencing the key role of structure and topology of such mixed systems [46].

In summary, we have explored here a new strategy to prepare fibrin-type I collagen hydrogels at high contents of both proteins. We could successfully achieve collagen fibrillogenesis within a fibrinogen network and use thrombin to obtain fibrin-based materials. However, our preliminary cellular studies indicate that a better tuning of collagen self-assembly is required to obtain mixed hydrogels with controlled architecture. The use of alternative neutralization pathways, such as using other buffers or an ammonia vapor treatment [47] or modification of the ionic strength conditions, are promising perspectives in this direction [48]. Beyond the two specific proteins investigated here, many other mixed biopolymer materials associating two gelling systems, such as gelatin-agarose [49] or collagen-chitosan [50], could benefit from the exploration of similar in-gel protocols. 

## 4. Materials and Methods

### 4.1. Formation of Fibrin-Type I Collagen Hydrogels

Collagen I was extracted from young rat tail tendons, and the final concentration was estimated by hydroxyproline titration, following published protocols [47,51]. The formation of mixed hydrogels went through three steps: (a) 500 μL of fibrinogen solution (40 mg.mL^−1^) at pH = 3.6 mixed with 500 μL collagen solutions (5, 10, 20 mg.mL^−1^) (pH = 3.6) were dispensed in 12 well-plates at 37 °C overnight to form the gels; 500 μL of fibrinogen solution (40 mg.mL^−1^) at pH = 3.6 was mixed with 500 μL acetic acid (0.5 mM) was used as control. (b) Citrate buffer solution (20 mM, sodium citrate-HCl, pH 7.2) was used to wash the gels until a neutral pH was achieved. (c) One milliliter of citrate solution containing 8 μL of 200 U thrombin was added to each well, and they were further kept at 37 °C overnight. 

### 4.2. Rheological Studies

Gels were tested using an MCR 302 rheometer from Anton Paar with a subsequent frequency sweep from 0.1 to 100 rad.s ^−1^ with 1% amplitude at room temperature. In order to test all matrices under similar conditions, the gap between the base and the geometry was chosen before each run so that a slight positive normal force was exerted on the gel during the measurement.

### 4.3. Scanning Electron Microscopy (SEM) Analysis

Hydrogels were fixed with 4% paraformaldehyde for 1 h and with 2.5% glutaraldehyde in cacodylate buffer for 1 h at 4 °C. Progressive dehydration was performed via successive water/ethanol baths with increasing ethanol content. After drying in supercritical CO_2_, samples were coated with a 10 nm gold layer and imaged using a Hitachi S-3400N microscope operating at 6–10 kV. 

### 4.4. Transmission Electron Microscopy (TEM)

Paraformaldehyde, glutaraldehyde (see above) and osmium tetroxide 4 wt% were used in sequence to fix the hydrogels. Hydrogels were then dehydrated using water/ethanol baths with increasing ethanol concentration, progressively impregnated with propylene oxide and incorporated in araldite resin prior to sectioning (Leica microtome) [52]. Sections were imaged by TEM on a Tecnai Spirit G2 operating at 120 kV (FEI Company, Eindhoven, The Netherlands). Images were recorded on a Gatan CCD Orius camera.

### 4.5. Cell Studies

C2C12 cells were grown in DMEM medium supplemented with 10% fetal bovine serum and 1% penicillin/streptomycin at 37 °C in a humidified incubator with 5% CO_2_ and passaged every 2 days; 1 mL of cell suspension at passage 14 containing 40,000 C2C12 cells were seeded on the pure fibrin and composite hydrogels prepared in 12-well plates. After 4, 5, 6 and 7 days, the medium was discarded and the Alamar Blue reagent (800 μL of a 0.1 mg.mL^−1^ solution, from Thermo Fisher) was added to the wells. After 4 h incubation at 37 °C, absorbance values at 570 nm and 600 nm were measured to obtain the reduction percentage of Alamar Blue. After 6 days of culture, gels were fixed with 4% PFA and cells permeabilized with 0.5% Triton X-100 for 15 min. After several rinsings with PBS solution, actin filaments were stained with Alexa Fluor 488 phalloidin and nuclei with 4′,6′-Diamidino-2-phenylindole (DAPI) solution. 

For differentiation studies, after 4 days of culture, the growth medium (DMEM medium supplemented with 10% fetal bovine serum and 1% penicillin/streptomycin) was replaced by 1 mL of differentiation medium (DMEM supplemented with 2% donor equine serum, 1% penicillin/streptomycin). After 7 additional days, gel fixation was performed as described above. Staining of myosin heavy chain was performed with MF-20 hybridoma mouse IgG2B primary antibody and Alexa Fluor 546 goat anti-mouse IgG2B secondary antibody (Invitrogen, Waltham, MA, USA). 

Gels were imaged with a fluorescence microscope (Axio Imager D.1, Zeiss, Jena, Germany) and processed using Image J software (Version 2.1.0/1.53c).

## Data Availability

Data are available upon reasonable request.
